# Supplementary material for: Sleep alterations as a function of 88 health indicators
Source: BMC Med. 2024 Mar 22;22:134. doi: 10.1186/s12916-024-03358-3 (PMC10960465; doi:10.1186/s12916-024-03358-3)
Supplement: Supplementary file 7 — Additional file 7. Supplementary table and figures. [file 12916_2024_3358_MOESM7_ESM.docx]

|  | SSRI | MAOI | TCA | TRAZ |
| --- | --- | --- | --- | --- |
| SSRI | 134 | 0 | 8 | 12 |
| MAOI | 0 | 4 | 0 | 0 |
| TCA | 8 | 0 | 46 | 3 |
| TRAZ | 12 | 0 | 3 | 72 |

**Supplementary table S1**. Antidepressant mono- and polytherapy in MrOS. The number of participants taking four types of antidepressant drugs (selective serotonin reuptake inhibitors [SSRI], monoamine oxidase inhibitors [MAOI], tricyclic antidepressants [TCA] and trazodone [TRAZ]) is reported in the diagonal. Off-diagonal cells contain the number of participants taking both drugs.


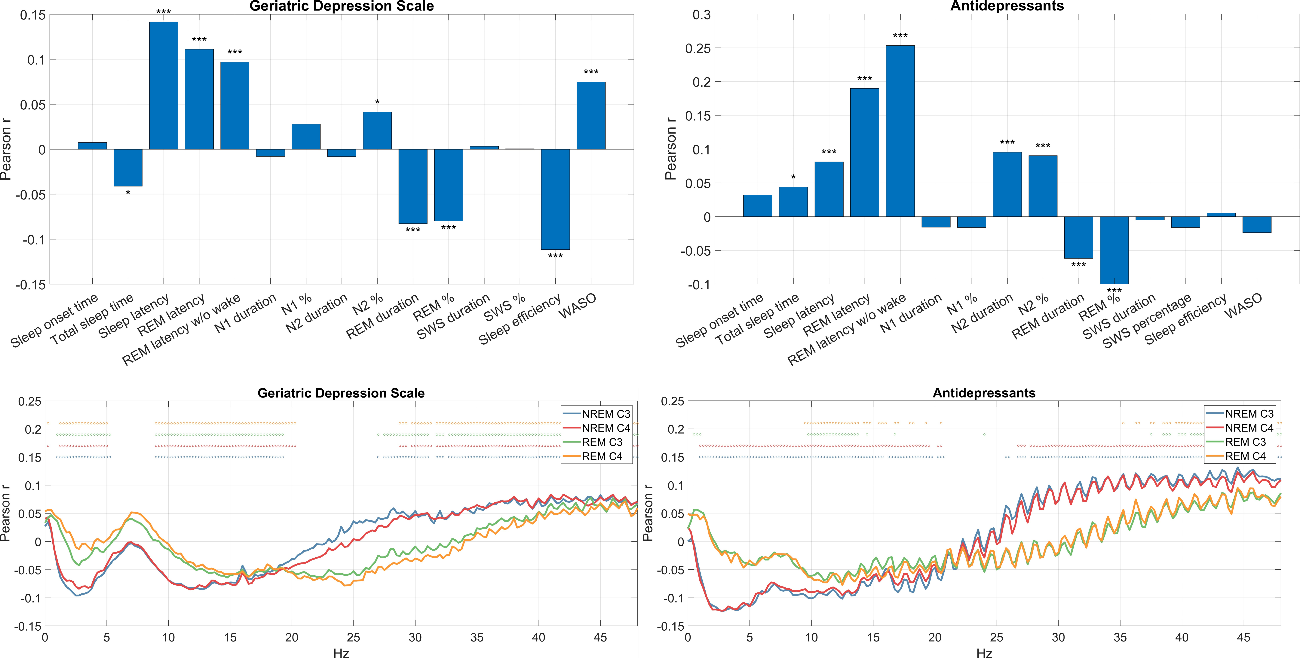


**Supplementary figure S1**. Correlations between sleep macrostructure (top panels) or EEG PSD (bottom panels) and Geriatric Depression Scale scores in antidepressant naïve participants (left panels) or antidepressant use (right panels).


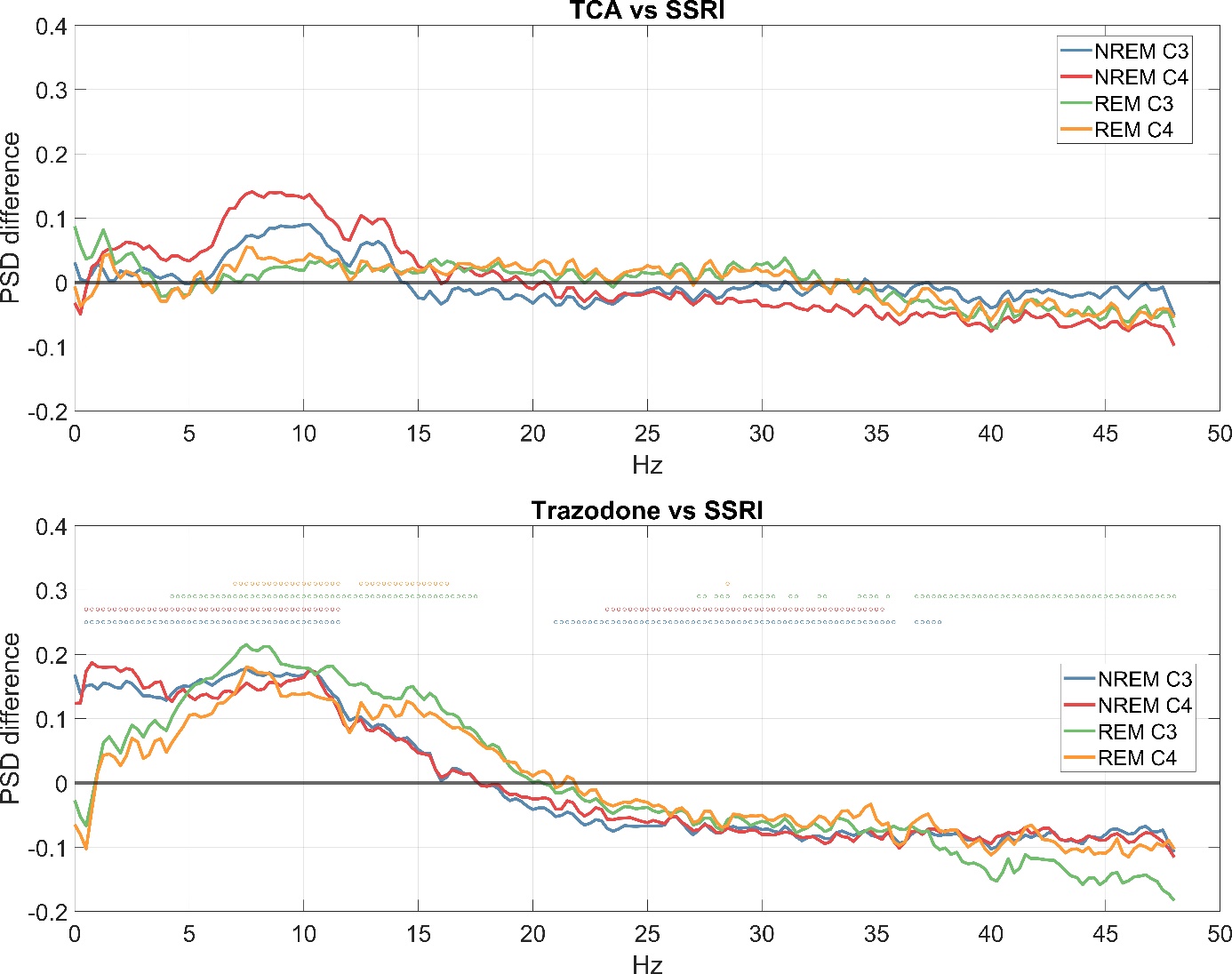


**Supplementary figure S2.** Comparing sleep EEG PSD in participant receiving different types of antidepressive treatment. Axis Y shows the expected PSD difference between SSRI users and TCA users (top) and trazodone users (bottom). Effects significant after correction for multiple comparisons are marked with a dot in the appropriate color above the lines. Note increased slow and decreased fast frequency activity in non-SSRI users. Analyses were restricted for participants undergoing antidepressive monotherapy. Participants reporting MAOI treatment were excluded due to low sample size (N=4).


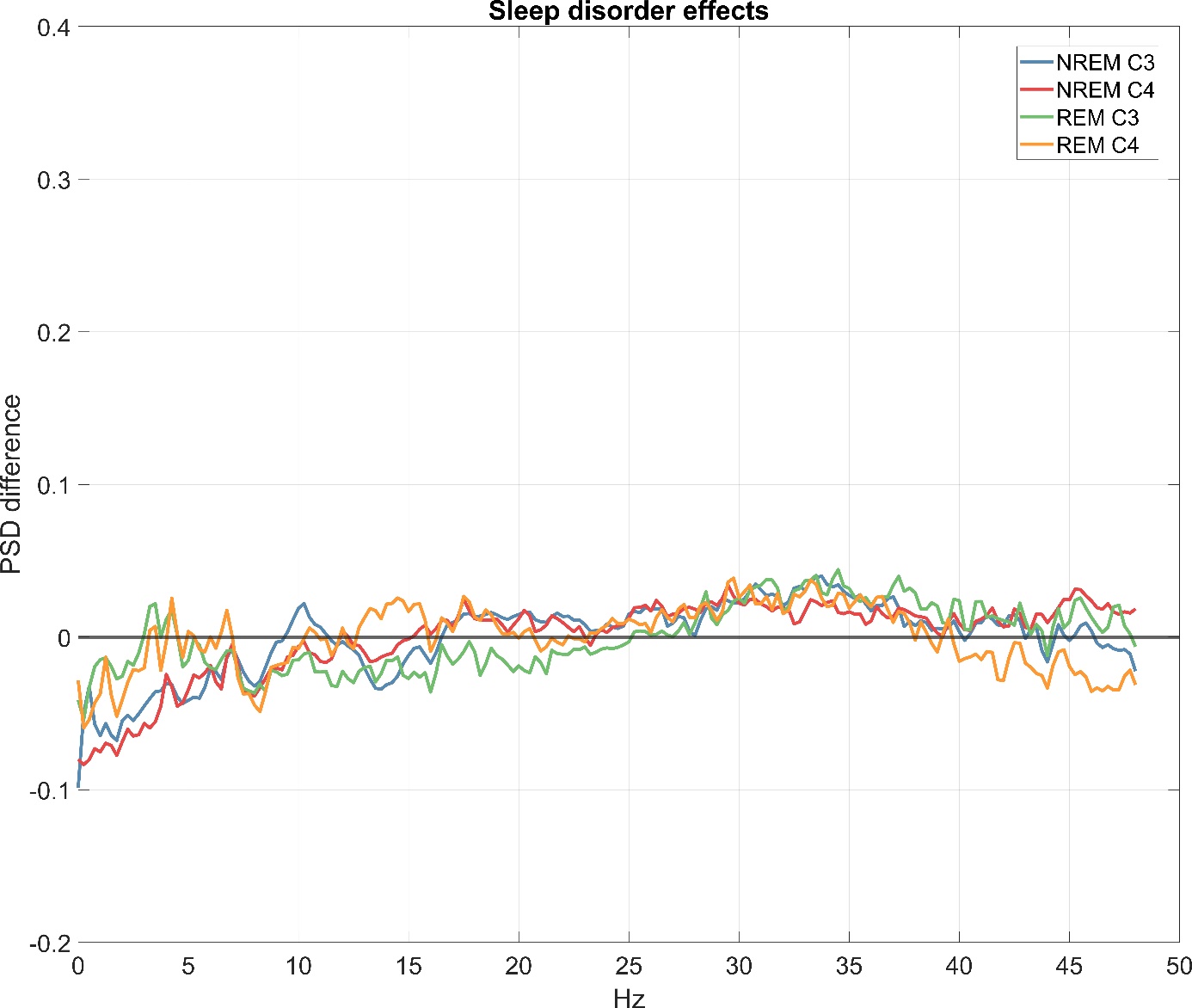


**Supplementary figure S3**. Comparing PSD in participants using benzodiazepine medication with (N=34) or without (N=100) sleep disorders. The chart shows log10 PSD differences between the two subgroups (axis Y) as a function of frequency (axis X) on both available channels in REM and NREM. No differences are significant even at the nominal p<0.05 level without correction for multiple comparisons.
